# *ABCB1* Variation and Treatment Response in AIDS Patients: Initial Results of the Henan Cohort

**DOI:** 10.1371/journal.pone.0055197

**Published:** 2013-01-25

**Authors:** Peng Zhu, Qian Zhu, Yilei Zhang, Xuejun Ma, Zizhao Li, Jie Li, Jiazhong Chen, Le Luo, Huijun Z. Ring, Brian Z. Ring, Li Su

**Affiliations:** 1 Key Laboratory of Molecular Biophysics of Ministry of Education, Huazhong University of Science and Technology, Wuhan, China; 2 Institute for Genomic and Personalized Medicine, School of Life Science and Technology, Huazhong University of Science and Technology, Wuhan, China; 3 School of Computer Science and Technology, Huazhong University of Science and Technology, Wuhan, China; 4 Institute for AIDS/STD Prevention and Control, Henan Center for Disease Prevention and Control, Zhengzhou, China; 5 State Key Laboratory for Molecular Virology and Genetic Engineering, National Institute for Viral Disease Control and Prevention, Chinese Center for Disease Control and Prevention, Beijing, China; 6 Health Department of Henan Province, Medical Science and Education Building, Zhengzhou, China; IPO, Inst Port Oncology, Portugal

## Abstract

HIV/AIDS has the highest mortality among infectious diseases in China. In ongoing efforts to alleviate this crisis, the national government has placed great emphasis on efforts in Henan province where HIV-infected former plasma donors in the 1990s contributed to AIDS becoming a public health crisis. Concomitant with a national initiative focusing the use of phamacogenetics for the better prediction of treatment response, we studied genetic variants with known pharmacokinetic phenotypes in a set of 298 HAART-treated (highly active antiretroviral therapy) patients infected with HIV from the Henan cohort. We measured the association of response to treatment, assessed as changes in CD4+ T cell counts after antiretroviral therapy, of five polymorphisms in four genes (*CYP2B6*, *ABCB1*/*MDR1*, *ABCG2,* and *ABCC4*) in which variation has been suggested to affect the pharmacokinetics of drugs commonly employed to treat HIV/AIDS. We show that genotyping for *ABCB1* variations (rs1045642 and rs2032582) may help predict HIV treatment response. We found variations in this gene have a significant association with outcome as measured by CD4+ T cell counts in a discovery subset (N = 197; odds ratio (OR) = 1.58; 95% CI 1.02–2.45), these results were confirmed in a validation subset of the cohort (N = 78; OR = 2.81; 95% CI 1.32–5.96). Exploratory analysis suggests that this effect may be specific to NVP (nevirapine) or 3TC (lamivudine) response. This publication represents the first genetic analysis in a continuing effort to study and assist the patients in a very large, unique, and historically significant HIV-AIDS cohort. Genotyping of AIDS patients for *ABCB1* variation may help predict outcome and potentially could help guide treatment strategies.

## Introduction

National efforts in China to stem the HIV epidemic have focused on Henan province as a key target area. In this province in the 1990’s, HIV-infected former plasma donors were a factor in the rise of the AIDS crisis in China [1]–[4]. To decrease the prevalence of HIV infection and mortality of AIDS patients in China, since 2003 the national government has offered a free anti-retrovirus treatment and prevention program. This program was piloted in 2002 in Henan province and has led to a significant decrease in AIDS related mortality, as well as increasing treatment coverage from very low levels to over 60% [5], [6]. However, a 2011 joint assessment by the Ministry of Health, UNAIDS and the WHO states that there remains 780,000 people with HIV infection in China, with approximately 154,000 cases of AIDS, in which there were about 28,000 AIDS-related deaths in 2011 [7]. Clearly even greater efforts must be applied to address this health crisis. Because of this continuing high mortality, a new national initiative focuses on how to use phamacogenetics in predicting treatment response [7].

Complete elimination of HIV is difficult to achieve with current clinical practices. In part due to resting infected T cells, HIV latency, and other factors, this residual infection contributes to therapy failure and resistant strains [8]. In a small study in Henan province, strains were found with a high level of resistance to non-nucleoside reverse transcriptase inhibitors (NNRTIs) and lower levels of resistance to nucleoside/nucleotide reverse transcriptase inhibitors (NRTIs); inadequate compliance to therapy appears to have been a primary factor for the development of this resistance [9]. The concentration of HIV infected patients in the Henan province compounds the epidemiological consequences of inadequately treated HIV infection. Consequently, since 2004 as part of an investigation by the Henan CDC, over 23,000 patients receiving standard HAART have their CD4+ T cell counts and viral load level monitored regularly. The very large Henan cohort is relatively genetically and socially homogenous with a high follow-up rate. Standard HAART for this cohort includes two NRTIs and one NNRTI or one protease inhibitor (PI) [10]. The expectation is that study of this large, unique, and well characterized cohort will identify useful pharmacogenetic associations to allow better treatment of HIV patients in China and elsewhere.

While there is consensus that monotherapy with available drugs is not effective to control HIV infection, the choice of drugs for combination therapy depends on several factors. The panel of drugs used to treat AIDS in China has been standardized and follows the national guidelines for the public antiretroviral therapy program. The antiretroviral NRTIs provided free of cost by the national government are: AZT, 3TC, d4T, ddI, ABC, and TDF; the NNRTIs are: EFV, NVP; and the PIs are: ATV, IDV, and LPV/r. Inclusion of AZT, NVP, 3TC or d4T, because of the ability of these drugs to bypass the blood-brain barrier, is common [11]. The preferred first-line therapy in treatment-naive adults is AZT or d4T +3TC+NVP, but as HAART usually contains dual NRTIs plus one NNRTI or PI there are many possible combinations. The choice of drugs employed is based on balancing patient tolerance and response, drug toxicity, as well as issues of cost and local supply. Additionally, the regimens will be modified if initial treatment is not effective or tolerated poorly. Decisions on which therapy choices to utilize are therefore directed in part by trial and error and the physician’s experience with different treatment strategies. Clearly, better prescriptive tools are needed.

Standard antiretroviral therapy doesn’t take the genetic variation of patients into account, contributing to varying treatment response among patients. In this study we explored the association of HAART response with five polymorphisms in four genes (*CYP2B6*, *ABCB1*/*MDR1*, *ABCG2,* and *ABCC4*) that have been shown to affect the pharmacokinetics of several standard HAART drugs and response to treatment. Polymorphisms in several genes, especially those coding proteins in drugs absorption, distribution, metabolism and excretion [12]–[15], have been implicated in altering the pharmacokinetics of some HAART drugs. *ABCB1* polymorphisms may predict virologic failure in response to EFV [16], variations in *CYP2B6* can predict pharmacokinetic aspects of EFV response [17], variants of the *ABCC4* gene (559G>T and 1460G>A) are associated with higher intracellular accumulation of AZT *in vitro* [18], and the *ABCG2* C421A allele is associated with the disposition of 3TC [19]. However, these variations are not utilized in standard clinical practice and their importance in a Chinese cohort is not known.

To study the association of selected SNPs with drug treatment outcome, the level of HIV infection was assessed by CD4+ T cell counts and viral load level in a cohort 298 HIV-infected Chinese patients. Participants either presented HIV positive serum confirmed by detection of HIV antibody, antigen and HIV nucleic acid or the patient was diagnosed as to having progressed to AIDS. The patients were deemed eligible for the study cohort if they received HAART and were living in four areas with high prevalence of HIV infection (Shenqiu, Shangcai, Weishi and Queshan counties) of Henan Province for more than six months. These patients were enrolled in a follow up investigation by the Henan CDC initiated in 2004; the participants’ viral load levels are monitored once per year and CD4+ T cell counts are monitored twice a year and the study is ongoing. This initial study is a pilot program with the goal of greatly extending the population that is being assessed genetically. The total cohort size in Henan Province is over 23,000. To prevent the transmission and decrease the mortality of HIV/AIDS in China, the government has established a comprehensive system, including HIV counseling and test, antiretroviral therapy, treatment monitor and a database of patients’ information of age, sex, treatment regimens, viral load and CD4 T cell counts each year. Based on this system, we plan for these initial results to be validated in the large Henan province cohort.

## Methods

### Study Participants

Random sampling was used to select a total of 298 HIV-infected Chinese patients living in four areas with high prevalence of HIV infection (Shenqiu, Shangcai, Weishi and Queshan counties) of Henan province for more than six months. These patients were enrolled in a follow up investigation by the CDC of Henan province from 2004 to 2011 and the participants’ viral load were regularly monitored once per year and CD4+ T cell counts were monitored twice a year. The median study period of the patients is 7.5 years, and the study is ongoing. The full criteria employed to determine inclusion into the study were: 1) the HIV serum positive status of the subject was confirmed by detection of HIV antibody, antigen and HIV nucleic acid according to the published “National Guidelines for Detection of HIV/AIDS” or the patient was diagnosed as to having progressed to AIDS; 2) the patient received HAART; 3) the treatment regimens had been recorded; 4) the patient viral load and CD4+ T cell count was assayed regularly and these records are available from the CDC of Henan province**.** Progression to AIDS in adults was diagnosed as any patient in WHO clinical stage 4 or for whom the CD4 count is less than 200 cells/mm^3^ or a CD4 percentage less than 15%. WHO clinical stage 4 is defined as suffering from severe associated diseases such as HIV wasting syndrome, pneumocystis pneumonia, recurrent severe bacterial pneumonia, chronic herpes simplex infection (orolabial, genital or anorectal of more than one month’s duration or visceral at any site) etc [20] This initial cohort is a pilot program, with the goal of greatly extending the studied population. This study is approved by the Henan Province Health Department and all participants provided written informed consent. Information on study participants’ personal characteristics, treatment regiment, and medical history was collected using a questionnaire.

### Genomic DNA Purification

Whole blood samples from participants were obtained by the local CDC. After erythrocyte lysis, white blood cells were separated and stored immediately at −80°C. Genomic DNA was isolated using the Wizard Genomic DNA Purification Kit (Promega, Madison, WI) following manufacturer’s instructions. The DNA was quantified with UV-vis spectrophotometer (NanoDropND-1000) and then diluted to working concentrations of 25 ng/µL.

### Genotyping

Five SNPs: *CYP2B6* 516G>T (rs3745274), *ABCB1* 3435T>A/C/G (rs1045642), *ABCB1* 2677T>G/A (rs2032582), *ABCG2* 421C>A (rs2231142), *ABCC4* 559G>T (rs11568658) were selected based on a literature review [19], [21]–[23]. The genotypes of all samples were determined by a high-throughput PCR-sequencing approach. The primers for PCR amplification and sequencing are listed in Table S1; the sequencing primer is the same as forward PCR primer. The genomic DNA samples were amplified using Premix ExTaq (2×, TaKaRa) in 96-well PCR plates (Axygen) with 25 µL of each PCR mixture composed of 12.5 µL Premix ExTaq (2×, TaKaRa), 1 µL genomic DNA (25 ng/µL), 1 µL of each primer (10 µM) and 9.5 µL ultra-pure water. The reaction was initially denatured at 95°C for 5 minutes followed by 40 cycles of 95°C for 30 seconds, 58°C for 30 seconds and 72°C for 30 seconds extension and completed by a final extension at 72°C for 10 minutes on Lifepro Thermal Cycler (Bioer). PCR products were purified according to the manufacturer’s protocol (GenMag Biotechnology Co., Ltd.). The purified PCR products were sequenced using MegaBace1000 (GE Healthcare).

### Statistical Analysis

Logistic regression models were used to estimate *P*-value, odds ratios (ORs) and 95% confidence intervals (95% CIs) for the ability of these genotypes to predict therapeutic response when a binary outcome measurement was used, and linear regression for the measurements using continuous values. CD4+ T cell counts were assessed twice per year from 2004 until 2011. In consultation with local physicians as to how CD4+ T cell counts is used to evaluate response to the aggressive early treatment strategy employed with these patients, the following scale was used: 0: CD4+ T cell counts were less than 250/mm^3^ at the final measurement; 1: counts consistently greater than 250/mm^3^ from the initial to final measurement; 2: counts improved from less than 250/mm^3^ in the initial measurement to greater than 250/mm^3^ in the final measurements. This was further simplified to a binary assessment of “no change” (0 or 1) and “improved” (2). These scales were determined prior to analysis. Genotype associations were assessed as additive, recessive, and dominant models. As there were two SNPs for *ABCB1*, these were also assessed using a simple additive model.

To determine independence of the alleles of other factors, namely age, sex and progression to AIDS, multivariate logistic regression modeling was employed. All results were considered significant if the *P*-value was less than 0.05. All *P*-values were 2-sided. Prior to study the cohort was randomly partitioned into a discovery (n = 215; subset with CD4 response data, n = 197) and validation (n = 83; subset with CD4 response data, n = 78) subsets, the validation subset was not used in the initial discovery analyses. The splitting of the cohort into discovery and validation cohorts was not employed when exploring association with specific treatments. Because the patient counts per genotype in the different treatment regimens was low for some subgroups a Fisher exact test was used to assess the distribution of patients in these analyses. The statistical analyses were performed using SPSS software (version19.0; IBM Corp, New York).

## Results

### Patient Characteristics

**Figure 1:**
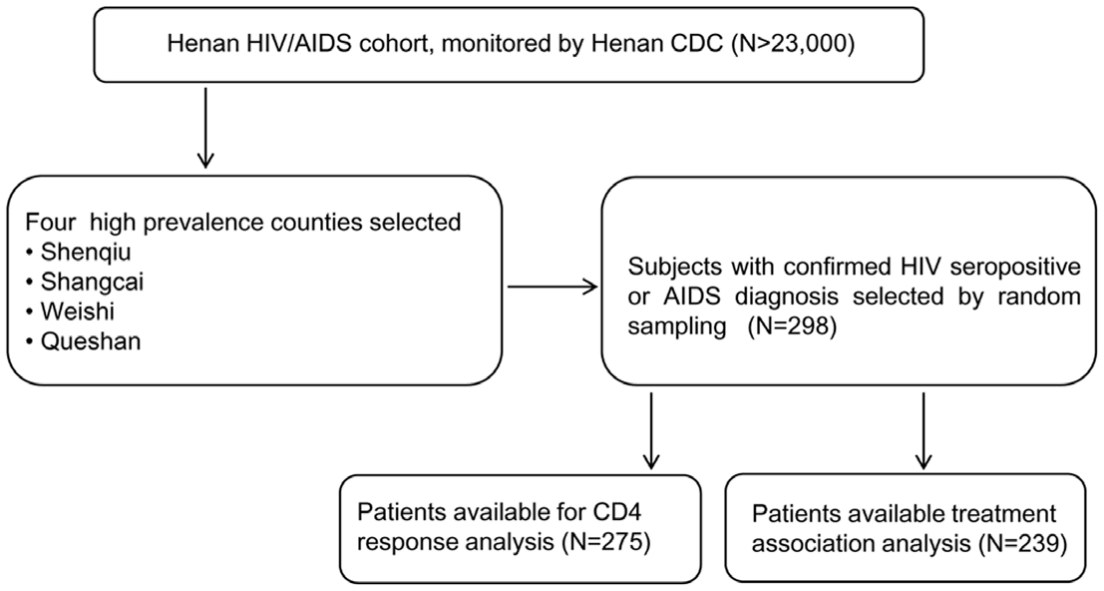
Flow chart of study participant selection.

The selection criteria of the patients are described in the materials and methods and the selection flowchart is shown in Figure 1. The demographic characteristics of the study patients are shown in Table 1. Median age at the initiation to the study was 45 years (range 23–70). Gender proportion of these patients is approximately equal (153/144 males/females). 87.6% of the patients received HAART because of low CD4+ T cell counts, high viral load, exhibiting WHO clinical stage 3 or 4, or volunteering to receive HAART even they were in WHO clinical stage 1 and 2.

**Table 1:** Demographic characteristics of the Henan cohort (N = 298).

Characteristics	Median (range)
Median age at diagnosis, years (range)	39 (16–66)
Median study time, years (range)	7.5 (1.6–12.3)
Median CD4 counts/µL, Year 1 (range)	334 (19–840)
Median CD4 counts/µL, Year 6 (range)	369 (8–1894)
Median viral load, RNA copies/ml (range)	350 (0–7600000)
Median time from probable infection until diagnosis, years	9.5 (0–18.4)
Median antiretroviral treatment, years (range)	6.4 (1.3–10.3)
Sex (M/F)	153/144
Fraction of patients who progress to AIDS	0.89

### Genotype Distribution and Allele Frequency

The five selected SNPs were genotyped by a high-throughput PCR-sequencing approach (Table S1). Each genotype distribution and allele frequency is summarized in Table 2. No deviation from Hardy-Weinberg equilibrium was detected for any allele assayed in this study.

**Table 2:** Distribution of candidate SNPs in studied subjects (N = 298).

GeneSymbol	Position	Allele	Frequency	Genotype	Subjects % (n)
*CYP2B6*	516G>T	G	0.84	GG	69.46% (207)
		T	0.16	GT	28.19% (84)
				TT	2.35% (7)
*ABCB1/* *MDR1*	3435T>C	T	0.43	TT	17.79% (53)
		C	0.57	CT	50.34% (150)
				CC	31.88% (95)
*ABCB1/* *MDR1*	2677T>G	T	0.61	TT	37.71% (112)
		G	0.39	GT	45.79% (136)
				GG	16.50% (49)
*ABCG2*	421C>A	C	0.69	CC	48.66% (145)
		A	0.31	AC	40.94% (122)
				AA	10.40% (31)
*ABCC4*	559G>T	G	0.87	GG	75.50% (225)
		T	0.13	GT	23.49% (70)
				TT	1.01% (3)

### Association of *ABCB1* with CD4+ T Cell Count Outcome

Response as measured by improvement in CD4+ T cell counts was significantly associated in the discovery subset with the combined *ABCB1* alleles in a recessive model (Table 3). The *ABCB1* SNPs (rs1045642, 3435T>C and rs2032582, 2677T>G) when assessed individually showed strong ORs but non-significance. The combined SNPs’ association was confirmed in the validation subset using the recessive model, and also was significant as an additive model. The two *ABCB1* SNPs were also significant when assessed individually in the validation set of patients. Outcome assessed on the three-level scale did not show a significant association with *ABCB1* in the discovery subset, but was significant in the validation set. *ABCB1* was also significant when assessed in the presence of age, sex, and whether the patient manifested AIDS (Table 4) in both the discovery and validation set of cases. In the discovery set, none of these additional clinical factors were significant in a multivariate model. These factors were also not significant when considered individually, neither were the factors: period from probable infection to diagnosis, time under treatment, and time under study (data not shown).

**Table 3:** Association with increase in CD4+ T cell counts.

			Allele dosage		Recessive model	
	Variation	N	OR (95% CI)	*P*	OR (95% CI)	*P*
DiscoverySet	*CYP2B6* 516G>T	197	0.79 (0.4–1.57)	0.498	0 (0–>10)	0.514
	*ABCB1* 3435T>C	197	1.41 (0.86–2.32)	0.165	1.94 (0.85–4.43)	0.113
	*ABCB1* 2677T>G	196	1.42 (0.87–2.34)	0.158	1.97 (0.98–3.96)	0.054
	*ABCG2* 421C>A	197	0.99 (0.58–1.68)	0.967	1.06 (0.32–3.47)	0.922
	*ABCC4* 559G>T	197	1.29 (0.63–2.65)	0.474	3.75 (0.22– >10)	0.354
	*ABCB1* *	197	1.25 (0.95–1.66)	0.107	1.58 (1.02–2.45)	0.040
ValidationSet	*CYP2B6* 516G>T	78	0.46 (0.12–1.79)	0.260	0.01 (0–>10)	0.724
	*ABCB1* 3435T>C	78	3.08 (1.21–7.84)	0.019	3.53 (1.01–12.41)	0.049
	*ABCB1* 2677T>G	78	2.59 (0.93–7.21)	0.067	5 (1.47–17)	0.011
	*ABCG2* 421C>A	78	0.79 (0.34–1.82)	0.573	0.38 (0.04–3.32)	0.375
	*ABCC4* 559G>T	78	1.75 (0.55–5.54)	0.336	0.01 (0–>10)	0.724
	*ABCB1* *	78	2.03 (1.13–3.64)	0.018	2.81 (1.32–5.96)	0.008

* *ABCB1* 3435T>C+*ABCB1* 2677T>G.

**Table 4:** Multivariate analysis.

	Discovery set (N = 182)		Validation set (N = 66)	
	OR (95% CI)	*P*	OR (95% CI)	*P*
ABCB1*	1.61 (1–2.59)	**0.048**	4.29 (1.56–11.83)	**0.005**
Age	0.99 (0.95–1.03)	0.641	1.01 (0.91–1.11)	0.884
Sex	1.28 (0.6–2.72)	0.519	0.09 (0.01–0.93)	**0.042**
AIDS	4.46 (0.56–>10)	0.154	>10 (0–>10)	0.743

ABCB1*: ABCB1 3435T>C+*ABCB1* 2677T>G.

### Association of *ABCB1* with Treatment Efficacy

**Figure 2:**
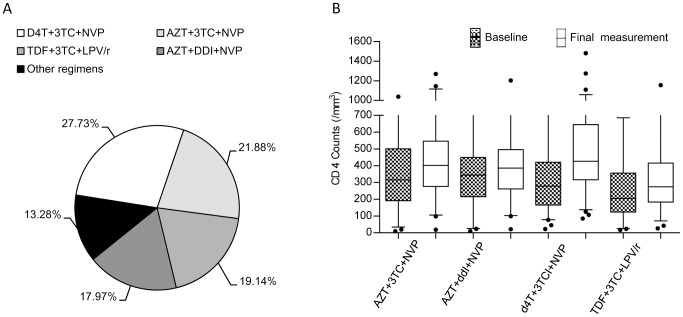
HAART regimens employed with this cohort. The four major regimens used within this cohort are shown (A), as well as the improvement in CD4 counts for each patient class (B) between the initial and final measurements. While there is observable variation in the overall outcome observed within the different treatment subsets, the differences were not significant.

A wide variety of treatment regimens has been employed with this cohort, using a number of different HAART combinations (Figure 2A and Table 5). There is variation in the overall outcome observed within different treatment subsets (Figure 2B), however in the subclasses of the explored regimens there were no significant differences. In an exploratory analysis between the SNPs and therapeutic response, a significant association of genotype and outcome for *ABCB1* and its individual SNPs was observed in all treatment regimens except for the subset of patients who received D4T plus any other drugs (Table 6). To ensure maximum power for this study, the splitting of the cohort into discovery and validation cohorts was not employed when exploring these associations with treatments. The association was strongest in those combinations that included NVP or 3TC. At least one of the two individual *ABCB1* SNPs showed a significant association with outcome in all tested regimens except for those containing D4T. Exploring this association with therapeutic choice further, we looked at the subsets of patients where a drug was excluded (Table S2). The association of *ABCB1* and outcome is nearly significant in patients who receive no D4T and just reaches significance in patients who do not receive AZT. However there is striking loss of significance in the patient subsets who do not receive 3TC or NVP; while patients using these two drugs exhibited the strongest association with outcome (Table 6). While not conclusive, this drug-specific effect suggests that the effect of the *ABCB1* variations on predicting outcome is mediated either through NVP or 3TC, or both. However the interaction of genotype with treatment is not significant (data not shown). Further studies are needed to validate these predictive associations.

**Table 5:** HAART Regimens.

	N	Regimen		N	Regimen
**3 drugs specified**	70	3TC NVP D4T	**One drug specified**	205	3TC + any other
	56	AZT 3TC NVP		178	NVP + any other
	49	3TC TD4 LVP		109	AZT + any other
	45	DDI AZT 3TC		83	D4T + any other
**2 drugs specified**	129	3TC NVP + any other		60	TDF + any other
	101	AZT NVP + any other		56	DDI + any other
	80	3TC D4T + any other		49	LPV + any other
	73	NVP D4T + any other		15	EFV + any other
	61	AZT 3TC + any other		8	(PV)R + any other
	60	3CT TDF + any other		1	(PV)N + any other
	51	DDI NVP + any other			

**Table 6:** Association analysis of SNPs and therapeutic response.

	Variation	3TC/NVP/other (N = 117)	D4T (N = 77)	AZT (N = 100)	3TC (N = 191)	NVP (N = 163)
**Allele dosage**	*CYP2B6* 516G>T	1.000	0.350	0.141	0.478	1.000
	*ABCB1* 3435T>C	**0.042**	0.167	0.452	**0.034**	**0.037**
	*ABCB1* 2677T>G	**0.013**	0.112	**0.009**	**0.025**	**0.003**
	*ABCG2* 421C>A	0.681	0.384	0.146	0.972	0.685
	*ABCC4* 559G>T	**0.020**	0.206	0.132	0.415	**0.026**
	ABCB1*	**0.041**	**0.228**	**0.177**	**0.048**	**0.020**
**Recessive model**	*CYP2B6* 516G>T	1.000	1.000	1.000	0.343	1.000
	*ABCB1* 3435T>C	**0.038**	0.116	0.303	**0.034**	**0.031**
	*ABCB1* 2677T>G	**0.005**	0.051	**0.009**	**0.008**	**0.001**
	*ABCG2* 421C>A	0.728	1.000	0.625	0.801	0.736
	*ABCC4* 559G>T	0.256	NA	0.200	1.000	0.215
	ABCB1*	**0.009**	0.053	**0.031**	**0.010**	**0.002**

ABCB1*: ABCB1 3435T>C + *ABCB1* 2677T>G.

## Discussion

In this study we discuss the findings of an initial genetic study of a historically important HIV cohort. This group of patients is unique both as a target of a very large national health initiative and for its uniform demographics, which should aid efforts to discover clinically applicable genetic associations. We found that improvement in CD4+ T cell counts over the course of treatment was significantly associated in a discovery subset with variation in *ABCB1*. This finding was upheld in a small validation subset of the cohort. Additionally, an exploratory analysis was performed to identify potential associations with treatment. Though the treatment regimens for this cohort were very diverse, we found preliminary evidence suggesting that the *ABCB1* variations may be mediating response to treatment with either NVP or 3TC, or both.

This is not the first association that has been made between ABCB1 and treatment, though identification of variation within this gene is currently not a standard clinical tool. ABCB1, also known as multidrug resistance protein 1 (MDR1), is a member of the MDR/TAP subfamily [24]. Expression of ABCB1 protein has been observed in several tissues as well as in epithelial cells of the blood–brain barrier [25], [26] and has been shown to transport a large variety of common pharmaceuticals [27]. There is considerable ethnic variation in the distribution of these variants; the distribution of the genotypes for the two *ABCB1* variants are significantly different between the European and Han HapMap populations (chi square *P*-value for rs1045642 and rs2032582 were *P*<0.001 and *P* = 0.002), and these variations may confound attempts at translating clinical results achieved in European populations to similar practice in China.

Many studies have assessed the potential associations of *ABCB1* polymorphisms with changes in expression, pharmacokinetics measurements, and drug response [16], [28]. Positive associations from these studies have proven difficult to validate. Several studies have specifically examined a potential association of genotype with outcome in HIV infected patients. In one of the initial studies of this kind, 96 patients treated with EFV or nelfinavir were examined for a relationship between the rs1045642 (3435 T>C) genotype and viral load or CD4+ T cell count. After 6 months of treatment it was found patients with a TT genotype had a significantly higher CD4+ count than the CT/CC genotypes but no significant association was found between genotype and viral load [28]. This is similar to the results found in this study, though the association found herein was with 3TC and NVP, there were too few EFV to assess the association. Other studies have not consistently confirmed this putative association [29]. For example, concentrations of lopinavir and ritonavir were not affected by variations in *ABCB1* [30]. Another randomized clinical trial of approximately 500 HIV patients assessed the association of the two *ABCB1* variants studied herein with outcome response to several drug therapies (patients received either EFV, nelfinavir or both, in conjunction with either ddI and D4T or AZT and 3TC). No significant association with plasma concentration was seen for either genotype. However a comparison of the TT genotypes vs. the combined CC/CT genotypes for the 3435 T>C variant was significant for the subgroups receiving EFV. Interestingly, this association was stronger in an analysis that controlled for race [16].

The difficulty in translating the well understood biochemical function of ABCB1 in transporting several drugs across cell membranes to consistent clinical findings could be due to an intrinsically small effect size. It is also possible that other factors make these studies difficult. HAART can employ several different drugs, in fact the use of several drugs is essential to help prevent the rise of resistant viral strains. However this makes strong associations studies difficult; the use of several drugs decreases the number of patients available to assess a single therapy combination. Additionally, it’s very likely drug-drug interactions are a significant confounding factor. This could be mediated through other enzymes, primarily the cytochrome P450 enzymes, or through ABCB1 itself. It has been shown in vitro that ABCB1 is induced in response to administration of NVP [31]. A solution to these potential problems is a large and uniform cohort. The large and well characterized Henan cohort, if expanded beyond this initial study, promises to be able to offer well powered studies in several individual drug subsets, and even for the most commonly employed drug combinations.

## Supporting Information

Table S1 **SNPs description and primers design.**
(DOCX)

Table S2 **Association analyses between SNPs and lack of drug in regimen.**
(DOCX)
