# Supplementary material for: ABCB1 Variation and Treatment Response in AIDS Patients: Initial Results of the Henan Cohort
Source: PLoS One. 2013 Jan 25;8(1):e55197. doi: 10.1371/journal.pone.0055197 (PMC3555879; doi:10.1371/journal.pone.0055197)
Supplement: Table S1 — SNPs description and primers design. (DOCX) [file pone.0055197.s001.docx]

**Table S1.** SNPs description and primers design

| **SNP ID** | **Gene Symbol** | **Primer** | **Primer sequence** |
| --- | --- | --- | --- |
| rs3745274 | *CYP2B6* 516G>T | F | TTACTGAGTGATGGCAGACAATCAC |
|  |  | R | CCGAATACAGAGCTGATGAGTGAA |
| rs1045642 | *ABCB1* 3435T>C | F | GCTGCTTGATGGCAAAGAAATAAAG |
|  |  | R | CCAGGCTGTTTATTTGAAGAGAGAC |
| rs2032582 | *ABCB1* 2677T>G | F | TCCTTCATCTATGGTTGGCAACTAA |
|  |  | R | CACATTCTTAGAGCATAGTAAGCAGTAG |
| rs2231142 | *ABCG2* 421C>A | F | TGCAGGTTCATCATTAGCTAGAACT |
|  |  | R | CATTACCTTGGAGTCTGCCACTTTA |
| rs11568658 | *ABCC4* 559G>T | F | CTTGTGCTTAGATCTGGTCTTGACA |
|  |  | R | GAAAGAGGGGTGTTTAATGCAATGA |

F: forward primer; R: reverse primer
