# Supplementary material for: ABCB1 Variation and Treatment Response in AIDS Patients: Initial Results of the Henan Cohort
Source: PLoS One. 2013 Jan 25;8(1):e55197. doi: 10.1371/journal.pone.0055197 (PMC3555879; doi:10.1371/journal.pone.0055197)
Supplement: Table S2 — Association analyses between SNPs and lack of drug in regimen. (DOCX) [file pone.0055197.s002.docx]

**Table S2.** Association analyses between SNPs and lack of drug in regimen

|  | | **Variation** |  | **no D4T (N=164)** | | **no AZT (N=141)** | | **no 3TC (N=50)** | | **no NVP (N=78)** | |
| --- | --- | --- | --- | --- | --- | --- | --- | --- | --- | --- | --- |
| **Allele dosage** | *CYP2B6* 516G>T | | | 0.243 | 0.309 | | 0.576 | | 0.741 | |  |
|  | *ABCB1* 3435T>C | | | 0.173 | 0.086 | | 0.517 | | 0.651 | |  |
|  | *ABCB1* 2677T>G | | | **0.027** | 0.269 | | 0.273 | | 0.403 | |  |
|  | *ABCG2* 421C>A | | | 0.616 | 0.342 | | 0.748 | | 0.720 | |  |
|  | *ABCC4* 559G>T | | | 0.781 | 0.813 | | 1.000 | | 0.675 | |  |
|  | *ABCB1** | | | 0.076 | 0.179 | | 0.445 | | 0.658 | |  |
| **Recessive model** | *CYP2B6* 516G>T | | | 0.591 | 0.572 | | NA | | 0.569 | |  |
|  | *ABCB1* 3435T>C | | | 0.070 | **0.034** | | 0.293 | | 0.387 | |  |
|  | *ABCB1* 2677T>G | | | **0.038** | 0.118 | | 0.370 | | 0.798 | |  |
|  | *ABCG2* 421C>A | | | 1.000 | 0.560 | | 1.000 | | 1.000 | |  |
|  | *ABCC4* 559G>T | | | 0.549 | 1.000 | | NA | | 1.000 | |  |
|  | *ABCB1** | | | 0.055 | **0.0499** | | 0.369 | | 0.445 | |  |

*ABCB1*: ABCB1* 3435T>C + *ABCB1* 2677T>G
